# Electrical impedance tomography applied to assess matching of pulmonary ventilation and perfusion in a porcine experimental model

**DOI:** 10.1186/cc7741

**Published:** 2009-03-05

**Authors:** Anneli Fagerberg, Ola Stenqvist, Anders Åneman

**Affiliations:** 1 Department of Anaesthesiology and Intensive Care, The Sahlgrenska Academy, Bla Straket 5, Gothenburg, SE 413 45, Sweden

## Abstract

**Introduction:**

Electrical impedance tomography (EIT) can be used to measure impedance changes related to the thoracic content of air and blood. Few studies, however, have utilised EIT to make concurrent measurements of ventilation and perfusion. This experimental study was performed to investigate the feasibility of EIT to describe ventilation/perfusion (V/Q) matching after acute changes of pulmonary perfusion and aeration.

**Methods:**

Six mechanically ventilated, anaesthetised pigs in the supine position were studied at baseline, after inflation of a balloon in the inferior caval vein (B_infl_) to reduce cardiac output and after an increased positive end-expiratory pressure (PEEP) of 20 cmH_2_O (PEEP20) to increase pulmonary aeration. EIT measurements were performed at the mid-thoracic level to measure the amplitude of impedance changes related to ventilation (Z_V_) and perfusion (Z_Q_), both globally and in four defined regions of interest (ROI) extending from the ventral to dorsal distance.

**Results:**

A largely parallel distribution of Z_V _and Z_Q _in all four ROIs during baseline conditions corresponded to a bell-shaped frequency distribution of Z_V_/Z_Q _ratios with only moderate scatter. B_infl _and PEEP20 with unchanged tidal volumes significantly increased the mismatch of regional Z_V _and Z_Q_, the scatter of Z_V_/Z_Q _ratios and the heterogeneity of the Z_V_/Z_Q _frequency distribution. Significant positive and negative correlations were demonstrated between fractional alveolar dead space (r^2 ^= 0.63 [regression coefficient]) and venous admixture (r^2 ^= 0.48), respectively, and the global Z_V_/Z_Q _ratio.

**Conclusions:**

EIT may be used to monitor the distribution of pulmonary ventilation and perfusion making detailed studies of V/Q matching possible.

## Introduction

Electrical impedance tomography (EIT) is a non-invasive, non-radiant, continuous technique that can be used to perform bedside measurements of impedance changes related to the thoracic content of air and blood [1,2]. Several clinical and experimental studies have demonstrated that EIT can be applied to monitor respiration and tailor the settings of mechanical ventilation [3-5]. The pulmonary circulation can also be monitored by EIT as reported both in normal conditions and during changes induced by disease or pharmacological interventions [6-10]. However, simultaneous measurements of pulmonary ventilation and circulation by EIT have previously been only scarcely reported [11-13]. The concurrent determination of ventilation-related and perfusion-related changes in impedance is complicated by the two signals differing in amplitude by up to an order of magnitude and being superimposed on each other with variable frequency harmonics.

We recently described how EIT can be used to monitor beat-to-beat changes in global pulmonary perfusion with adequate precision during acute variations in stroke volume related to changes in cardiac preload of pulmonary and extra-pulmonary origin [14]. This was performed by applying short periods of apnoea. The present investigation was designed to assess the matching of ventilation and perfusion, as reflected by the ventilation/perfusion (V/Q) ratio derived from EIT measurements. In addition to global measurements, the left and right lung ventilation and perfusion were studied separately and regionally in order to provide a comprehensive assessment of overall and regional V/Q matching.

The EIT algorithm generates values for impedance changes relative to a baseline measurement in every element of the reconstructed tomographic slice [15]. Significant changes in baseline conditions, for example sudden reductions in blood volume or the occurrence of pneumothorax or haemothorax, may confound the interpretation of impedance changes in absolute terms at different time points [16]. Therefore, the relation and distribution of impedance changes within and between defined regions of interest for any given time point, as utilised in the present experimental design, may provide a more robust estimate of ventilation and circulation in different parts of the lung. The V/Q results may potentially be helpful in optimising ventilator and haemodynamic therapy, taking into account the complex cardiorespiratory interactions, and could significantly extend the clinical applicability of EIT.

The aim of the experiment was to investigate the feasibility of EIT to describe the dynamic distribution of pulmonary ventilation and perfusion at resting conditions and during interventions designed to generate a wide range of V/Q ratios. The application of 20 cmH_2_O of positive end-expiratory pressure (PEEP) was used to primarily increase pulmonary aeration with secondary reduction of cardiac preload. Inflation of a balloon in the inferior caval vein was used to acutely reduce cardiac preload with minimal effects on pulmonary ventilation.

We hypothesised that combined measurements by EIT of lung ventilation and circulation could be used to assess V/Q matching including the extremes of venous admixture and wasted ventilation. It was further hypothesised that V/Q ratios by EIT would correlate to shunt and dead space fractions calculated using standard formulae.

## Materials and methods

Six Swedish Landrace pigs (weighing 32 to 34 kg) were included in the study following approval by the Ethics Committee for Animal Experiments at the University of Göteborg. Animal care conformed to the principles set forth in the *Guide for the Care and Use of Laboratory Animals *[17]. A limited dataset from two of the animals included in this study has previously been reported [14].

### Anaesthesia

After an overnight fast with free access to water, animals were premedicated using ketamine (13 mg/kg intramuscularly) and midazolam (2 mg/kg intramuscularly). Induction of anaesthesia was performed using an intravenous bolus of 10 mg/kg sodium pentobarbital and anaesthesia was then maintained using a combination of sodium pentobarbital (beginning at 25 mg/kg/hour and gradually lowered to 10 mg/kg/hour) and fentanyl (2.5 μg/kg/hour). Animals were ventilated via a tracheostomy with 40% supplemental oxygen in a volume-controlled mode with no end-inspiratory pause with PEEP set at 5 cmH_2_O (Servo 300, Siemens-Elema, Sweden), I:E ratio 1:2 and a tidal volume of 10 ml/kg resulting in an end-inspiratory pressure less than 35 cmH_2_O throughout the study. Normocapnia (5.0 ± 0.8 kPa (mean ± standard deviation)) was maintained as gauged by end-tidal capnometry and repeated arterial blood-gas analyses. Isotonic 2.5% glucose was administered intravenously at 10 ml/kg/hour and all animals were kept normothermic (38 to 39°C) using heating blankets.

### Preparation

A pulmonary artery catheter (7.5 F Opticath™, Abbott Laboratories, Chicago, IL, USA) was inserted via the right internal jugular vein to monitor cardiac output (as the mean of triplicate thermodilution measurements randomly throughout the respiratory cycle) and to obtain mixed venous blood samples. An arterial catheter was inserted in the left femoral artery to monitor mean arterial pressure and to obtain arterial blood samples. A Fogarty embolectomy catheter (Edwards Lifesciences Services GmbH, THB 32080810F, Unterschleissheim, Germany) was placed via the right femoral vein in the inferior caval vein with its inflatable balloon just below the diaphragm.

### Electric impedance tomography

A 16-electrode silicon belt was positioned at the mid-thoracic level and connected to the EIT monitor (EIT Evaluation Kit 2, Dräger Medical, Lübeck, Germany). A tomographic image of impedance changes was created every 100 ms (10 Hz) by the application of an electrical current of 5 mA and 50 kHz to the electrodes. The image was reconstructed in a 32 by 32 pixel matrix. The tomographic slice by EIT is about 5 cm thick [1], thus well in excess of the reported cranio-caudal displacement of the lungs during ventilation [18,19].

**Figure 1:**
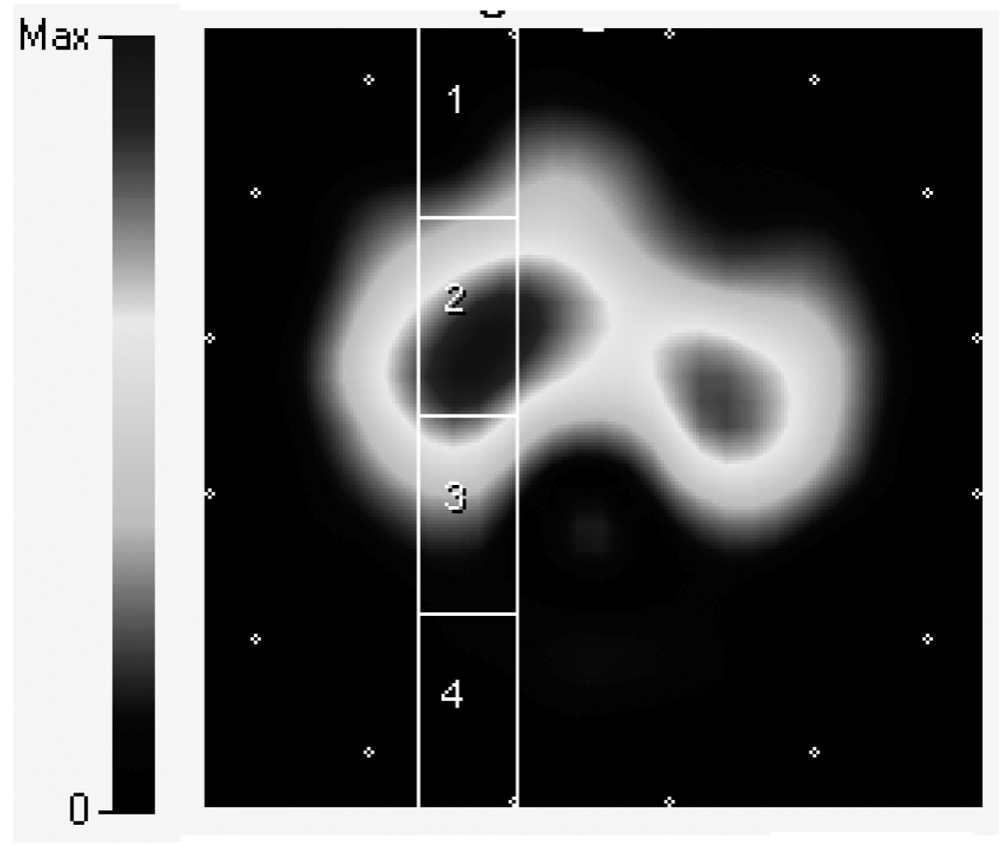
Electrical impedance tomography image at end-inspiration illustrating for the right lung the setting of the four regions of interest. Region of interest (ROI) 1 = ventral; ROI 2 = mid-ventral; ROI 3 = mid-dorsal; ROI 4 = dorsal. Note that ROI 1 and ROI 4 encloses relatively minor parts of aerated lung tissue. The medial border of the ROIs was set just lateral to the cardiac contour identified during apnoea.

The unfiltered EIT signal was recorded and converted to ASCII files using dedicated EIT software (Draeger EIT Data Review, Dräger Medical, Lübeck, Germany). Four equally sized regions of interest (ROIs) of eight by four pixels each, together extending the full ventral to dorsal distance, were set either along a left or right axis going through the lung fields, taking care not to include the cardiac area (Figure 1). Measurements of impedance changes within the four ROIs thus represented the ventral (ROI 1), mid-ventral (ROI 2), mid-dorsal (ROI 3) and dorsal (ROI 4) parts of either the left or right side. All ASCII files were analysed off-line using custom-made software (MATLAB 7.4.0.287 (R2007a), Mathworks Inc., Natick, MA, USA) to calculate the amplitude of impedance (Z) changes during ongoing ventilation (Z_V_, five to eight breaths) and during a short apnoea (Z_Q_, 15 to 25 pulse beats). The reconstruction algorithm for the EIT signal is based on impedance differences in every pixel measured and is thus dimensionless [15]. Both Z_V _and Z_Q _were recorded in arbitrary units (AU).

### Experimental design

All animals were investigated in the supine position at steady state during baseline and following either the increase of PEEP from 5 to 20 cmH_2_O (PEEP20) or the inflation of the Fogarty balloon catheter (B_infl_). At each point in the protocol, EIT recordings were made during ongoing ventilation and during a short end-expiratory pause. Arterial and mixed venous blood samples were simultaneously collected and the inspiratory fraction of oxygen and the end-tidal carbon dioxide (AS/3 Anaesthesia Monitor, GE Healthcare, Solna, Sweden) were noted. The arterial and mixed venous blood samples were immediately analysed using an automated blood gas analyser set to analyse pig blood samples (ABL700, Radiometer, Copenhagen, Denmark). The partial pressures of oxygen and carbon dioxide, oxygen saturation and haemoglobin content were entered into the experimental documentation file.

### Calculations and statistical analyses

All values are given as the mean and standard deviation. Both Z_V _and Z_Q _were multiplied by the respiratory rate and the heart rate, respectively, to provide a common format of AUs per minute. The relative distribution of Z_V _and Z_Q _to each ROI was calculated as the proportion of the global, cumulative sum of ROI 1 to 4 for the left and right lung, respectively, and the Z_V_/Z_Q _ratio in each ROI was calculated by dividing the relative values for Z_V _and Z_Q_. Venous admixture (Q_s_/Q_t_) was calculated according to the standard formula based on pulmonary capillary, arterial and mixed venous oxygen contents. Fractional alveolar dead space (V_D_/V_T_) was estimated by dividing the arterial to end-tidal carbon dioxide gradient by the arterial carbon dioxide tension [20]. Changes in Z_V_, Z_Q_, their distribution and the Z_V_/Z_Q _ratios were analysed by paired student's t-test. The correlations between Z_V_/Z_Q _ratios by EIT and Q_s_/Q_t _and V_D_/V_T _were evaluated using Pearson linear correlation, using pooled data from baseline, PEEP20 and B_infl _in each animal. Statistical significance was set at a p < 0.05. All statistical analyses were performed using Prism 5 for Mac OSX (GraphPad Software, Inc., San Diego, CA, USA).

## Results

Baseline cardiac output was 3.0 ± 0.8 L/minute and decreased to 1.8 ± 0.3 L/minute during PEEP20 and to 1.4 ± 0.3 L/minunte during B_infl_, corresponding to stroke volumes of 27 ± 4 mL at baseline, 14 ± 4 mL during PEEP20 and 12 ± 4 mL during B_infl_. Mixed venous oxygen saturation decreased from 70 ± 3% at baseline to 41 ± 9% during interventions. Baseline Q_s_/Q_t _was 11 ± 2% and decreased to 5 ± 1% during PEEP20, whereas baseline V_D_/V_T _increased from 9 ± 4% to 27 ± 11% during B_infl_.

The greatest impedance amplitudes for Z_V _and Z_Q _at baseline were observed in the mid-ventral region (ROI 2) with no significant differences between the left and right lung. B_infl _reduced Z_Q _in both left and right ROI 2, although an increase was noted in left ROI 1. PEEP20 also decreased Z_Q _in the left and right ROI 2 and increased Z_V _in ROI 3.

**Figure 2:**
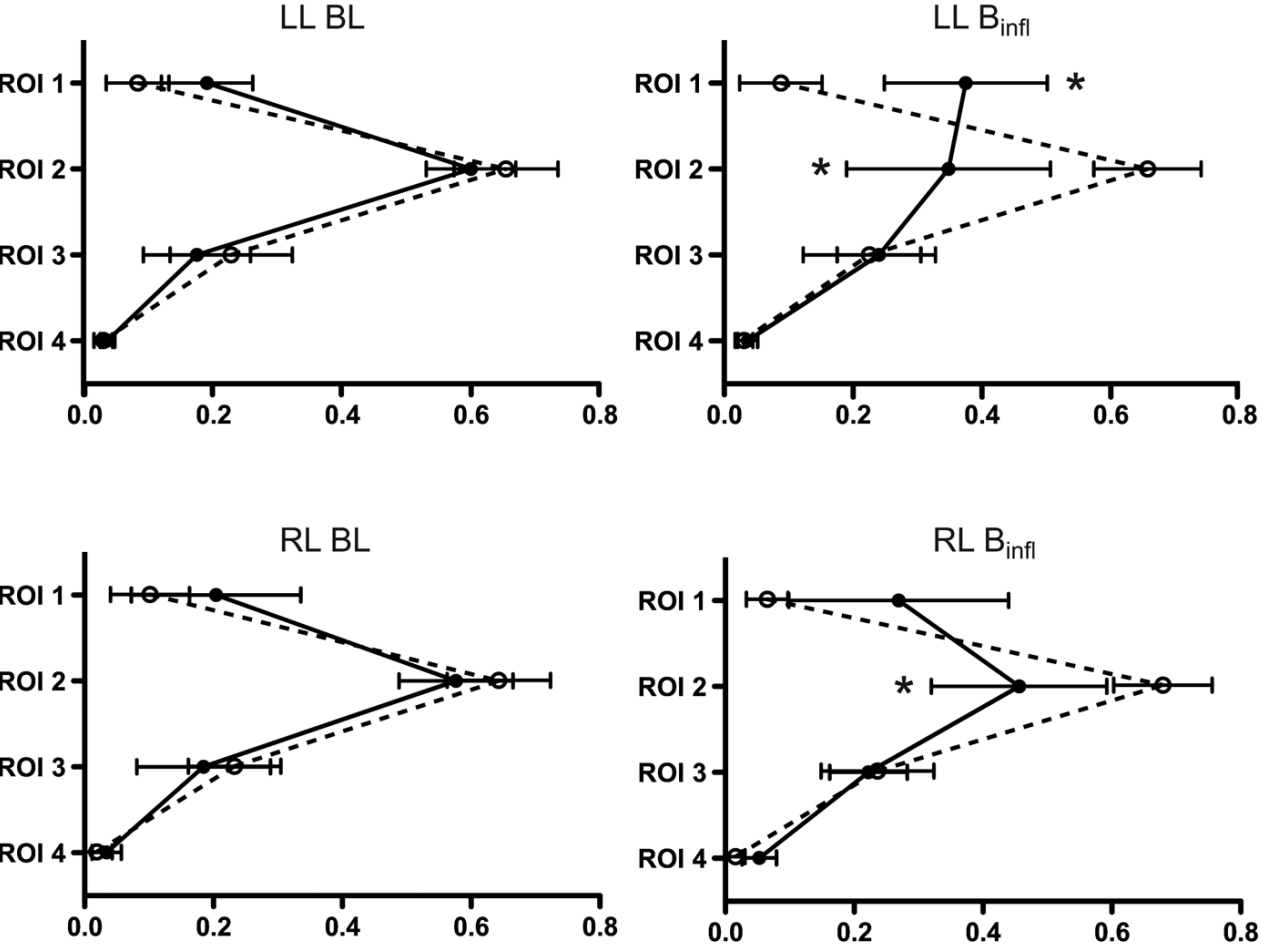
Relative distribution of Z_V _and Z_Q _within ROI 1 to 4 for the left and right lung at baseline and following balloon inflation. * denotes significant difference (p < 0.05) for Z_Q _compared with baseline. Relative distribution given as a percentage. Z_V _= ventilation-induced change in thoracic impedance; dotted line. Z_Q _= perfusion-induced change in thoracic impedance; solid line. Region of interest (ROI) 1 = ventral; ROI 2 = mid-ventral; ROI 3 = mid-dorsal; ROI 4 = dorsal. B_infl _= balloon inflation; BL = baseline; LL = left lung; RL = right lung.

**Figure 3:**
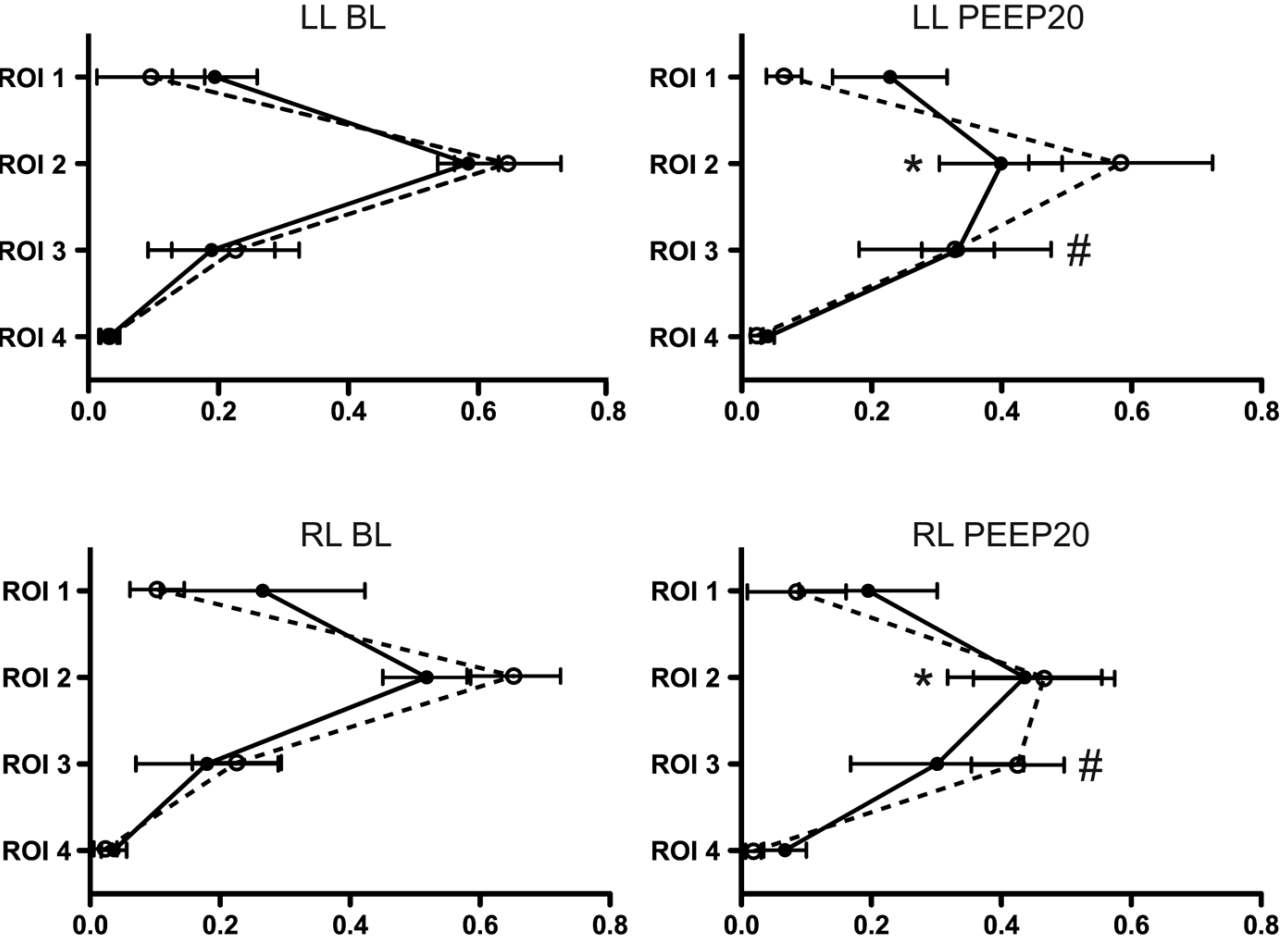
Relative distribution of Z_V _and Z_Q _within ROI 1 to 4 for the left and right lung at baseline and following the application of PEEP 20. * denotes significant difference (p < 0.05) for Z_Q _compared with baseline. # denotes significant difference (p < 0.05) for Z_V _compared with baseline. Relative distribution given as a percentage. Z_V _= ventilation-induced change in thoracic impedance; dotted line. Z_Q _= perfusion-induced change in thoracic impedance; solid line. Region of interest (ROI) 1 = ventral; ROI 2 = mid-ventral; ROI 3 = mid-dorsal; ROI 4 = dorsal. BL = baseline; LL = left lung; PEEP20 = the application of 20 cmH_2_O of positive end-expiratory pressure; RL = right lung.

The relative distribution of Z_V _and Z_Q _between the individual ROIs in relation to the total, cumulative signal in ROI 1 to 4 is illustrated in Figures 2 and 3. At baseline conditions, a parallel distribution of Z_V _and Z_Q _was observed, with the greatest proportion of ventilation and perfusion appearing in ROI 2 followed by ROI 3 and then ROI 1, while only a minor, although equal, fraction was observed in ROI 4. B_infl _decreased the proportion of Z_Q _distributed to ROI 2 in both the left and right lung and increased the proportional Z_Q _in the left ROI 1. No changes were observed in the distribution of Z_V _between the ROI 1 to 4. Hence, Z_V _increased in relation to Z_Q _in ROI 2 bilaterally, although the reverse was observed in the left ROI 1 (Figure 2). PEEP20 increased the proportion of Z_V _in ROI 3 bilaterally while Z_V _in ROI 2 tended to decrease. A reduction of relative Z_Q _was observed in ROI 2 and thus Z_V _increased in relation to Z_Q _in ROI 2 and with a similar trend in ROI 3 (Figure 3).

**Figure 4:**
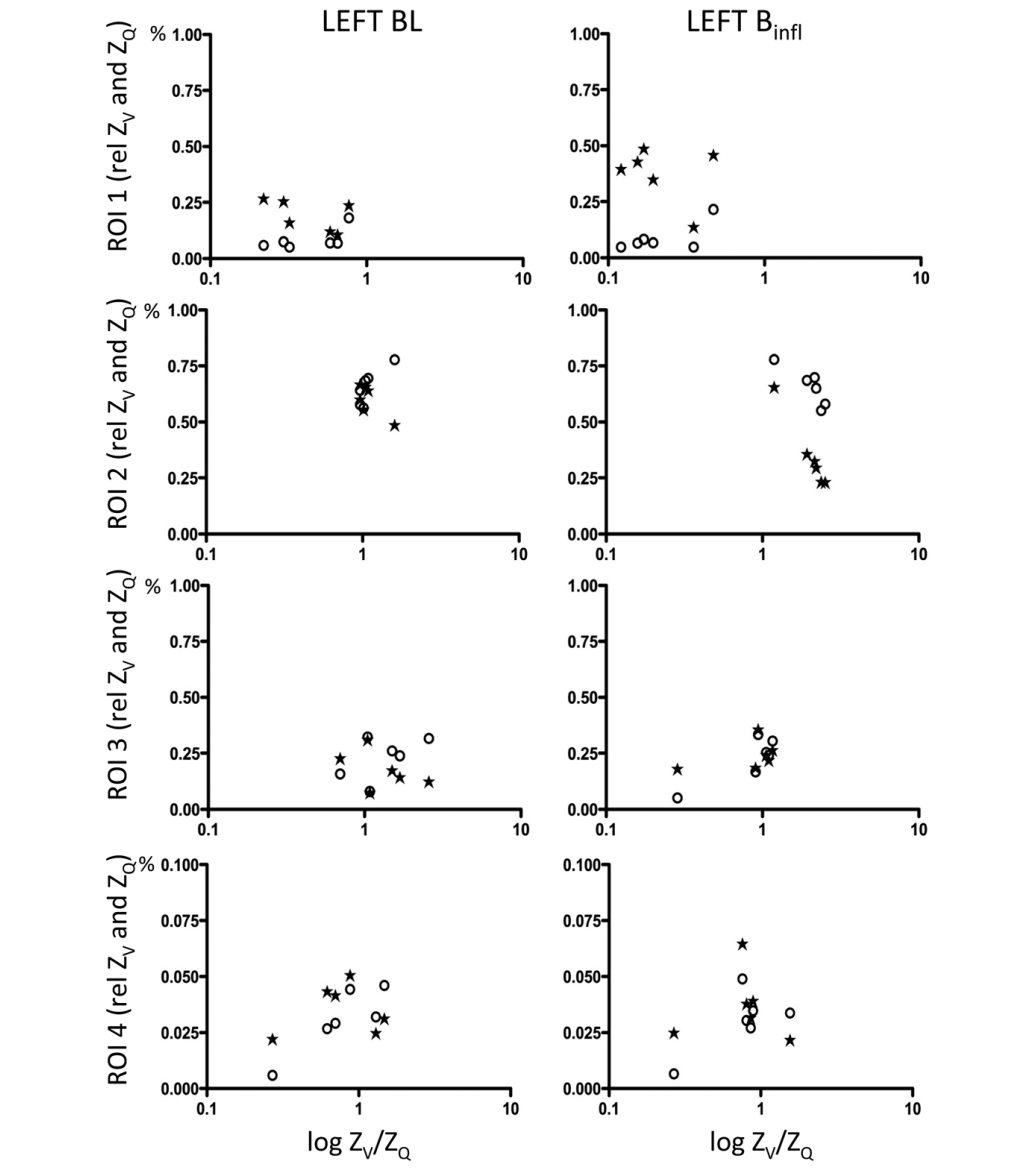
Scatterplots of relative Z_V _and Z_Q _within ROI 1 to 4 for the left lung at baseline and following balloon inflation in relation to the relative Z_V_/Z_Q _ratio. Z_V _= ventilation-induced change in thoracic impedance; open circle. Z_Q _= perfusion-induced change in thoracic impedance; solid stars. Values presented as a proportion of global Z_V _and Z_Q_. Region of interest (ROI) 1 = ventral; ROI 2 = mid-ventral; ROI 3 = mid-dorsal; ROI 4 = dorsal. B_infl _= balloon inflation; BL = baseline.

**Figure 5:**
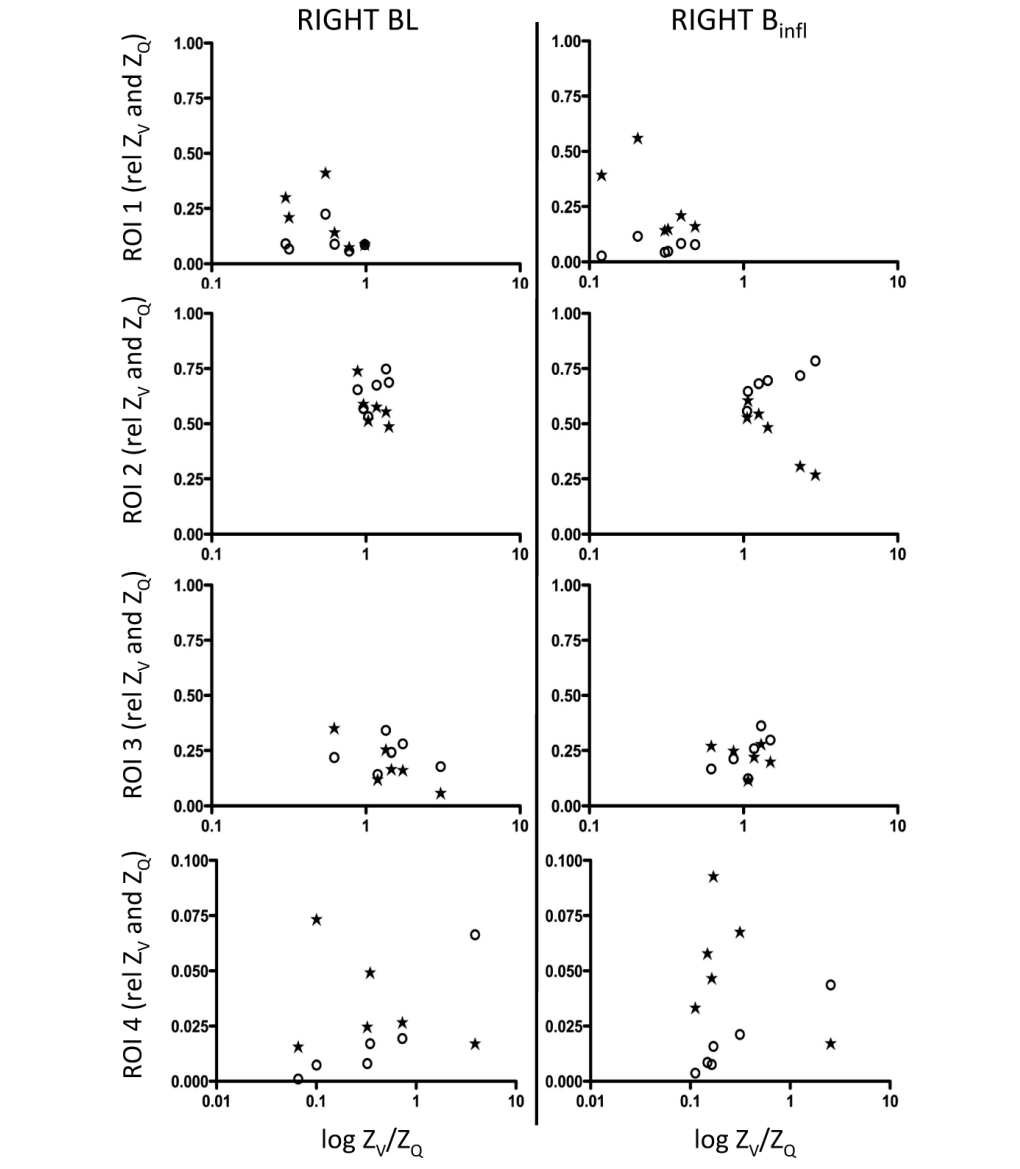
Scatterplots of relative Z_V _and Z_Q _within the ROI 1 to 4 for the right lung at baseline and following balloon inflation in relation to the relative Z_V_/Z_Q _ratio. Z_V _= ventilation-induced change in thoracic impedance; open circle. Z_Q _= perfusion-induced change in thoracic impedance; solid stars. Values presented as a proportion of global Z_V _and Z_Q_. Region of interest (ROI) 1 = ventral; ROI 2 = mid-ventral; ROI 3 = mid-dorsal; ROI 4 = dorsal. B_infl _= balloon inflation; BL = baseline.

**Figure 6:**
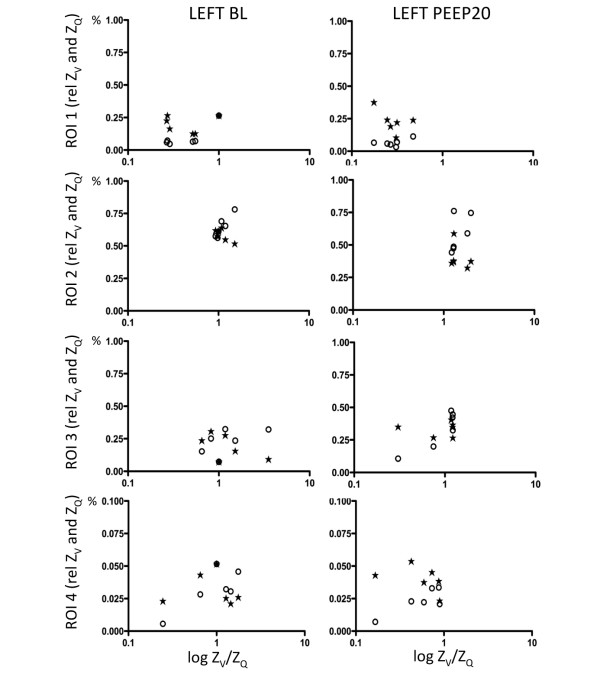
Scatterplots of relative Z_V _and Z_Q _within the ROI 1 to 4 for the left lung at baseline and following PEEP20 in relation to the relative Z_V_/Z_Q _ratio. Z_V _= ventilation-induced change in thoracic impedance; open circle. Z_Q _= perfusion-induced change in thoracic impedance; solid stars. Values presented as a proportion of global Z_V _and Z_Q_. Region of interest (ROI) 1 = ventral; ROI 2 = mid-ventral; ROI 3 = mid-dorsal; ROI 4 = dorsal. BL = baseline; PEEP20 = the application of 20 cmH_2_O of positive end-expiratory pressure.

**Figure 7:**
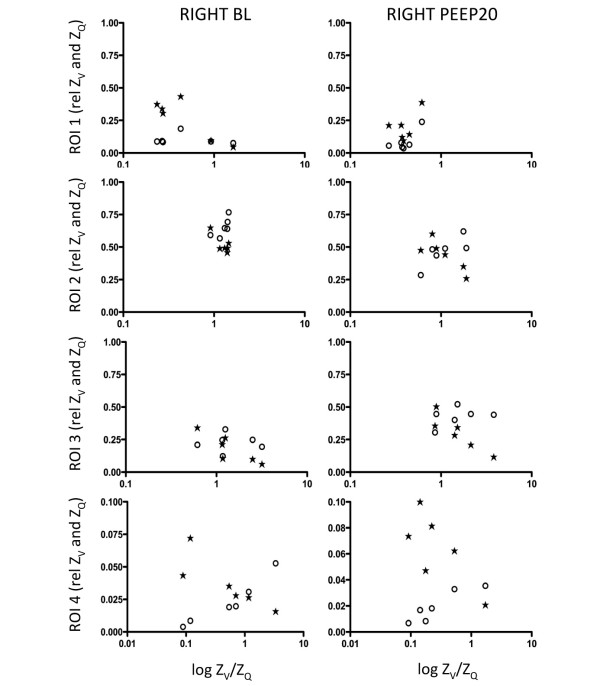
Scatterplots of relative Z_V _and Z_Q _within the ROI 1 to 4 for the right lung at baseline and following PEEP20 in relation to the relative Z_V_/Z_Q _ratio. Z_V _= ventilation-induced change in thoracic impedance; open circle. Z_Q _= perfusion-induced change in thoracic impedance; solid stars. Values presented as a proportion of global Z_V _and Z_Q_. Region of interest (ROI) 1 = ventral; ROI 2 = mid-ventral; ROI 3 = mid-dorsal; ROI 4 = dorsal. BL = baseline; PEEP20 = the application of 20 cmH_2_O of positive end-expiratory pressure.

The calculated Z_V_/Z_Q _ratios were plotted against Z_V _and Z_Q _before and after B_infl _(Figures 4 and 5) and PEEP20 (Figures 6 and 7). At baseline, a close to ideal Z_V_/Z_Q _ratio of 1 was observed in ROI 2 and similarly in ROI 3 and 4 although with increased scatter, while a trend towards venous admixture was noted in ROI 1. B_infl _increased the Z_V_/Z_Q _ratio in ROI 2 indicating dead space ventilation while increased venous admixture appeared in ROI 1. Changes in ROI 3 and 4 did not reach statistical significance because of considerable scatter. Following PEEP20, Z_V_/Z_Q _ratio increased in ROI 2 consistent with dead space ventilation and a similar trend was noted in ROI 3 while venous admixture appeared in ROI 1. No significant changes appeared in ROI 4 as a result of persistent scatter.

**Figure 8:**
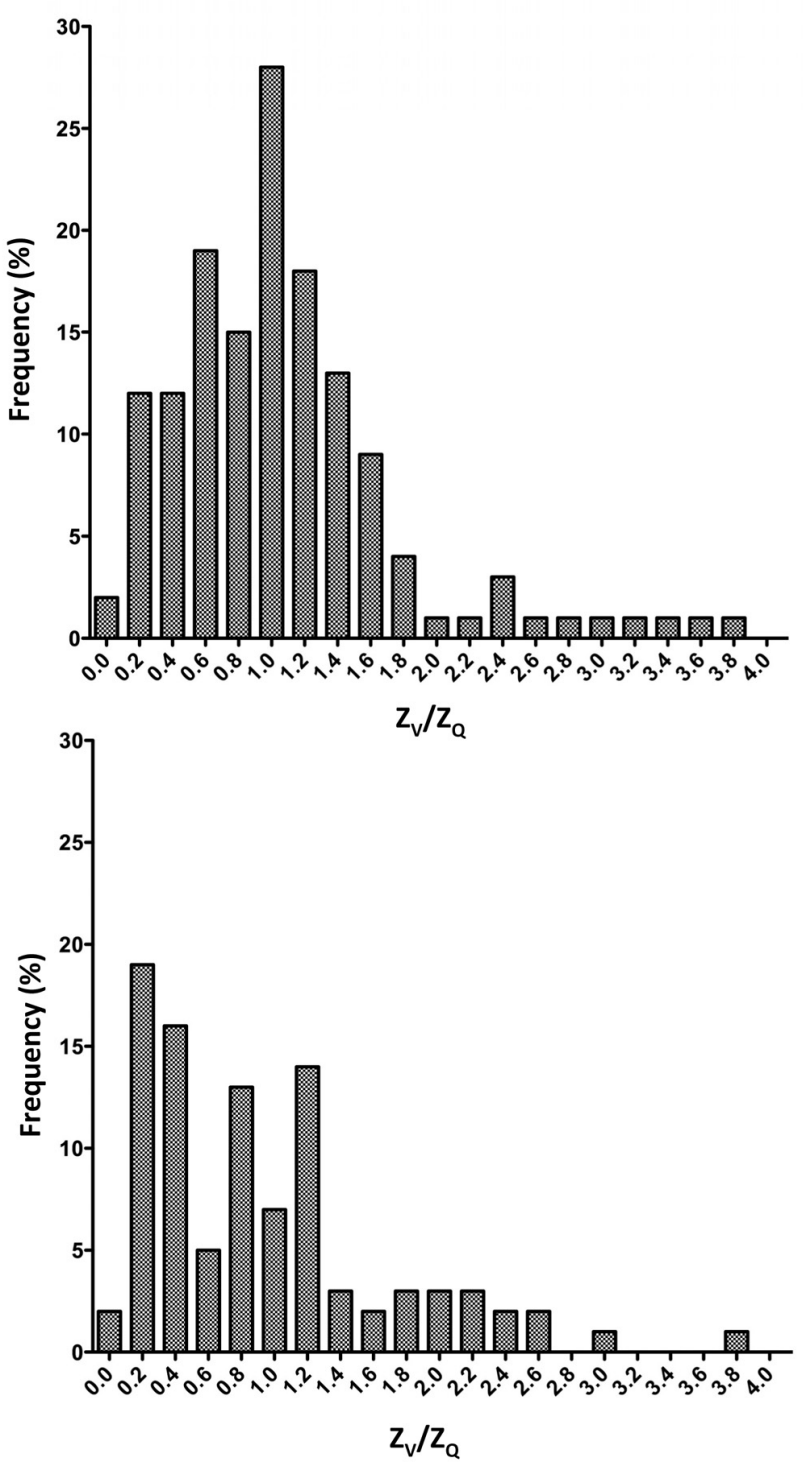
Frequency distribution of Z_V_/Z_Q _ratios at baseline (top) and following balloon inflation or increase to PEEP20 (bottom). PEEP20 = the application of 20 cmH_2_O of positive end-expiratory pressure; Z_Q _= perfusion-induced change in thoracic impedance; Z_V _= ventilation-induced change in thoracic impedance.

**Figure 9:**
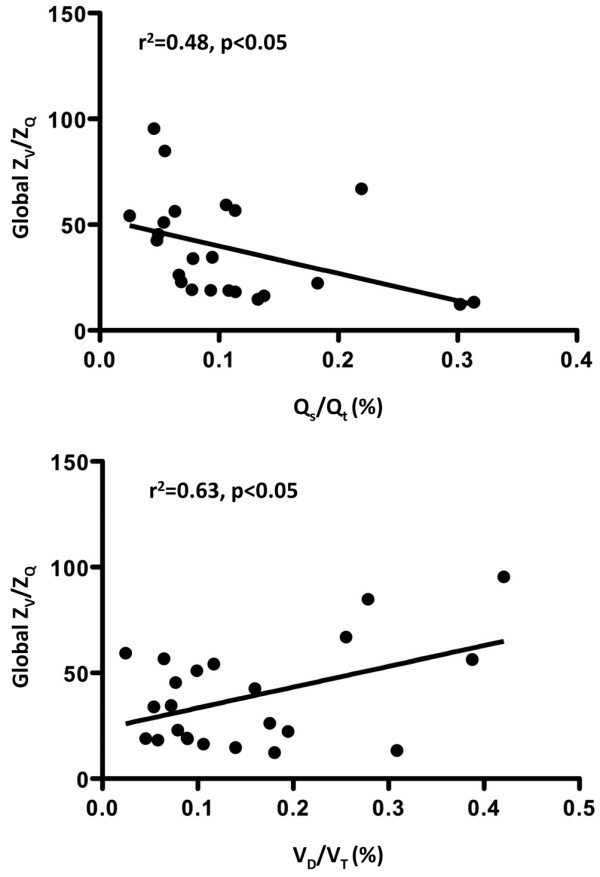
Linear regression plots for the global Z_V_/Z_Q _ratio versus the Q_s_/Q_t _(top) or the V_D_/V_T _(bottom). Q_s_/Q_t _= venous admixture; V_D_/V_T _= fractional dead space; Z_Q _= perfusion-induced change in thoracic impedance; Z_V _= ventilation-induced change in thoracic impedance.

The frequency distribution of all EIT Z_V_/Z_Q _ratios calculated at baseline demonstrated a Gaussian distribution with a mean of 1.1 (95% confidence interval = 0.96 to 1.2). This distribution was markedly changed into a non-Gaussian pattern during B_infl _and PEEP20 with a mean of 0.86 (95% confidence interval = 0.78 to 1.1; Figure 8). The calculated Z_V_/Z_Q _ratios based on global EIT correlated to venous admixture (r^2 ^= 0.48 [regression coefficient]) with a negative slope (y = -194 × +67) and to fractional dead space (r^2 ^= 0.63) with a positive slope (y = 92 × +32; Figure 9).

## Discussion

This study demonstrated the use of EIT to study pulmonary ventilation and perfusion in parallel. Using an experimental model to generate a wide range of stroke volumes for a constant tidal volume, we were able to investigate the dynamic relation between Z_V _and Z_Q_. By defining four ROIs along the ventral to dorsal axis for each of the left and right sides separately, an extensive dataset of Z_V_/Z_Q _ratios was collected. A largely parallel proportion of Z_V _and Z_Q _in all four ROIs during baseline conditions corresponded to a bell-shaped frequency distribution of Z_V_/Z_Q _ratios with only moderate scatter. Acute changes in stroke volume, established by inflating a balloon in the inferior caval vein or increasing PEEP from 5 to 20 cmH_2_O, while tidal volume was maintained, significantly increased the mismatch of regional, proportional Z_V _and Z_Q_, the scatter of Z_V_/Z_Q _ratios and the heterogeneity of the Z_V_/Z_Q _frequency distribution. Significant positive and negative correlations were demonstrated between fractional alveolar dead space and venous admixture, respectively, and the global Z_V_/Z_Q _ratio.

Although an expanding body of evidence supports the use of EIT to monitor pulmonary ventilation and perfusion separately, only few studies have been published on their contemporaneous measurements. Deibele and colleagues, using similar EIT equipment, focused on technical aspects to separate the cardiac and respiratory related signals in two healthy, spontaneously breathing volunteers [12]. Kunst and colleagues investigated left-to right division of ventilation and perfusion in 14 spontaneously breathing patients scheduled for lobectomy or pneumectomy for lung cancer [13]. Frerichs and colleagues studied pulmonary perfusion in three pigs using a hypertonic saline solution as an electrical impedance contrast agent [21]. Thus, this study extends previous observations by adding regional separation and V/Q matching during anaesthesia and mechanical ventilation. Venous admixture was assumed to be an inherent feature of the model using anaesthetised animals studied in the supine position with low PEEP. The balloon inflation mimics a situation of acute onset hypovolaemia and increased PEEP is commonly applied in mechanically ventilated patients to prevent or reduce lung collapse.

The method to assess pulmonary perfusion used in this study has been described previously [14] but warrants some comments. Global Z_Q _represents an approximation of stroke volume but, like any other method, has an inherent bias and variability, although within clinically acceptable limits. The physiology behind cardiac-related changes in Z_Q _is still imperfectly understood and, for example, the relative influence of large pulmonary vessels compared with pulmonary capillaries needs further research. Furthermore, Z_Q _relates to a change in air/blood content by the stroke volume rather than flow velocity and no separation of the latter in terms of alveolar capillary flow or shunt flow can be made.

The definition of ROI is an essential point in the data analysis. In this study, four equally sized ROIs were set by default, covering the full anterioposterior distance and the Z_Q _and Z_V _signals were analysed off-line using a stand-alone computer algorithm. It remains possible that setting the four ROIs within the pulmonary boundaries of the individual EIT image might have resulted in other Z_V _and Z_Q _amplitudes. In particular the most dorsal ROI 4 may be less representative because of the low impedance signal in this region [22]. The default full anterioposterior approach using equally sized ROIs was chosen in the design of this study to minimise operator-related bias. Initial pilot data indicated that placing the ROIs individually in each analysis significantly increased inter- and intraobserver variability.

The distribution of ventilation and perfusion at baseline differed along the ventral to dorsal distance with the greatest proportion delivered to the mid-ventral followed by the mid-dorsal regions. This is in line with the concept of gravitational influence on distribution and appeared even more clearly if all four ROIs were individually adjusted to the apparent pulmonary field of the EIT image (data not shown). Importantly, the distribution of Z_V _and Z_Q _were very closely matched, as would be expected in stable baseline conditions. Balloon inflation significantly reduced Z_Q _in ROI 2 leading to apparent dead space ventilation in this region, while an apparent redistribution of Z_Q _towards ROI 1 in the left lung would contribute to venous admixture. However, it should be noted that ROI 1 represents a moderate volume of tissue with the heart and lungs in close proximity.

The spatial resolution of EIT is inferior to that of other imaging techniques, for example computed tomography, and cannot exclusively separate impedance changes originating from the cardiopulmonary border. It is thus possible that the recorded increase in Z_Q _in ROI 1 during balloon inflation results from an increased cardiac contribution rather than an increase in pulmonary blood flow. Increased PEEP to 20 cmH_2_O shifted ventilation towards the more dorsal region, as previously reported [5]. At the same time, Z_Q _was reduced, leading to a relative increase in dead space ventilation. Thus, the well-matched distribution of Z_Q _and Z_V _was partially lost following the interventions. The relative distribution of Z_Q _and Z_V _highlights general changes in V/Q matching. In order to gain a more detailed picture, the individual relative Z_Q _and Z_V _were plotted against the Z_V_/Z_Q _ratio. This approach to illustrate V/Q matching is less prone to artefacts induced by the selection of ROIs because all data comes from within the same ROI, as opposed to the distribution plot that relates one ROI to all others.

Again, ventilation and perfusion were closely matched at baseline, although a scatter of Z_V_/Z_Q _ratios appeared in the most ventral and dorsal parts of the lung. Balloon inflation increased the scatter in most ROIs and increased dead space ventilation in ROI 2, consistent with the acute reduction of blood flow in this region. PEEP ventilation increased dead space in ROI 2 and 3, whereas venous admixture appeared in ROI 1, which is in agreement with the dorsal redistribution of ventilation. Furthermore, the Z_V_/Z_Q _plots demonstrated the heterogeneity including both wasted ventilation and perfusion that turned out as significant correlations to shunt and dead space calculated independently of EIT. The relative ease of operation, low cost and lack of invasive procedures and radiation of EIT compares favourably with other methods to assess V/Q matching such as radionuclide scanning [23] or multiple inert gas elimination technology [24].

This was primarily a feasibility study of EIT to determine V/Q relations, and as such has some important limitations. The results are based on a small group of animals and pooled data from multiple observations in each animal was used, which might exaggerate the statistical significance demonstrated. All animals were without lung pathology and thus the results regarding PEEP should not be directly extrapolated to the clinical context when PEEP is applied during mechanical ventilation for respiratory failure. The interruption of ventilation when measuring perfusion-related impedance changes excludes the effects of tidal volume changes on pulmonary circulation. Although the acquisition of EIT data could be achieved on-line within minutes, the off-line analyses were more time consuming and would not be directly available at the bedside. No comparisons were made to other methods of assessing V/Q matching such as radionuclide scanning [23] or the multiple inert gas technique [24], and hence data on precision and bias of EIT in this respect is lacking.

## Conclusion

We conclude that EIT might be used to monitor the distribution of pulmonary ventilation and perfusion making detailed studies of V/Q matching possible. The technique holds substantial potential although further work is necessary to position EIT in the context of clinical critical care.

## Key messages

• EIT can be applied to monitor Z_V _and Z_Q _in isolated ROIs.

• A largely parallel, proportional distribution of Z_V _and Z_Q _in all ROIs during baseline conditions corresponded to a bell-shaped frequency distribution of Z_V_/Z_Q _ratios with only moderate scatter.

• Acute changes in stroke volume, established by inflating a balloon in the inferior caval vein or increasing PEEP from 5 to 20 cmH_2_O, while tidal volume was maintained, significantly increased the mismatch of regional, proportional Z_V _and Z_Q_, the scatter of Z_V_/Z_Q _ratios and the heterogeneity of the Z_V_/Z_Q _frequency distribution.

• Significant positive and negative correlations were demonstrated between fractional alveolar dead space and venous admixture, respectively, and the global Z_V_/Z_Q _ratio.

• EIT might be used to monitor the distribution of pulmonary ventilation and perfusion making detailed studies of V/Q matching possible.

## Abbreviations

AU: arbitrary units; B_infl_: the inflation of a Fogarty balloon in the inferior caval vein; EIT: electrical impedance tomography; PEEP20: the application of 20 cmH_2_O of positive end-expiratory pressure; Qs/Qt: venous admixture; ROI: region of interest; V_D_/V_T_: fractional alveolar dead space; V/Q: ventilation/perfusion ratio; Z_Q_: perfusion-induced change in thoracic impedance; Z_V_: ventilation-induced change in thoracic impedance.

## Competing interests

OS has received lecturing reimbursements from Dräger Medical. AF and AÅ declare that they have no competing interests.

## Authors' contributions

All authors equally contributed to the design, data acquisition, data analysis and manuscript preparation. All authors have read and approved the final manuscript.
